# Red-seaweed biostimulants differentially alleviate the impact of fungicidal stress in rice (*Oryza sativa* L.)

**DOI:** 10.1038/s41598-022-10010-8

**Published:** 2022-04-09

**Authors:** Sahana N. Banakar, M. K. PrasannaKumar, H. B. Mahesh, P. Buela Parivallal, M. E. Puneeth, Chirag Gautam, D. Pramesh, T. N. Shiva Kumara, T. R. Girish, Sailaja Nori, Shrikumar Surya Narayan

**Affiliations:** 1 Department of Plant Pathology, University of Agricultural Sciences, Bangalore, India; 2 Department of Genetics and Plant Breeding, College of Agriculture, V. C. Farm, Mandya, India; 3 Department of Plant Pathology, Agriculture University, Kota, India; 4 Rice Pathology Laboratory, All India Coordinated Rice Improvement Programme, Gangavathi, University of Agricultural Sciences, Raichur, India; 5 Sea6 Energy, Pvt Ltd, Center for Cellular and Molecular Biology, (C-CAMP), Bangalore, India

**Keywords:** Plant molecular biology, Plant stress responses

## Abstract

Red seaweed-derived biostimulants facilitate plant health and impart protection against abiotic stress conditions by their bioactive compounds and plant nutrients. The potency of red seaweed biostimulants (LBS6 and LBD1) on rice cv. IR-64 in response to fungicides induced stress was investigated in this study. Foliar application of LBS6 maintained the stomatal opening and leaf temperature under the fungicidal stress condition. Reactive Oxygen Species (ROS) such as hydrogen peroxide and superoxide radicals were significantly reduced in LBS6-treated stressed plants. After applying seaweed biostimulants, ROS production was stabilized by antioxidants viz*.,* CAT, APX, SOD, POD, and GR. LBS-6 application increased the Ca^+^ and K^+^ levels in the stressed plants, which perhaps interacted with ROS and stomatal opening signalling systems, respectively. In the rice plants, fungicidal stress elevated the expression of stress-responsive transcriptional factors (*E2F*, *HSFA2A*, *HSFB2B*, *HSFB4C*, *HSFC1A*, and *ZIP12*). A decline in the transcript levels of stress-responsive genes was recorded in seaweed treated plants. For the first time, we present an integrative investigation of physicochemical and molecular components to describe the mechanism by which seaweed biostimulants in rice improve plant health under fungicidal stress conditions.

## Introduction

Rice (*Oryza sativa* L.) is an important cereal crop and a staple food for a large population of the world, especially in South and Southeast Asia. The major constraints in rice production are biotic and abiotic stresses^1^. Each year, these stresses cause large-scale crop losses, which are expected to escalate as climate change is projected. Fungicides are crucial strategies for managing fungal diseases in rice since they are the most destructive worldwide. The excessive use of fungicides causes fungicidal stress. Besides, fungicide application also has more significant impact on the residual levels in the crop. Reducing pesticides in agriculture is essential to minimize the environmental impact and improve the sustainability of agricultural systems^2^. As a result, agricultural practices have evolved towards organic, sustainable, or environmentally friendly practices. Organic molecules such as seaweeds may be used as one of the strategies to minimize pesticide use. Unlike pesticides, these organic compounds are non-toxic and non-polluting^3^. Seaweed extracts have been shown to promote plant growth, minimize abiotic stresses by regulating molecular, physiological, and biochemical processes^4^. In general, abiotic stress responses involve the production of Reactive Oxygen Species (ROS), ionic imbalance, altered Ca^2+^, K^+^ signalling, stomatal behaviour and leaf temperature as well as the induction of heat stress factor genes (HSFs) and other transcription factors (TFs) which limit the growth and productivity of plants^4–6^. Seaweed extracts are emerging as commercial formulations to boost tolerance by targeting multiple stress pathways. Seaweeds are red, green and brown macroalgae representing 10% of marine productivity^2^. The red and brown algae make up the vast majority of seaweed formulations. Tropical red seaweeds are macroalgae that can accelerate plant metabolism and boost plant efficiency. The presence of phytohormones and several organic molecules acting as compatible solutes has been attributed to the beneficial effects of seaweed extracts^7,8^. High concentrations of phenolic compounds with antioxidant properties that protect against stress-induced ROS can be used to improve stress tolerance^9^. Previously, red seaweed was also shown to enhance the expression of MAP kinase genes, stress-responsive transcription factor WRKY, antioxidative catalase and Superoxide dismutase (SOD) under abiotic stress conditions in wheat^10^. Though plant stress is often viewed at the level of whole plant, initial responses to stress occur at the leaf level. Higher leaf temperatures and stomatal closure may affect plant processes directly by injuring the photosynthetic apparatus^11^. Calcium and potassium are essential nutrients that affect several physiological and biochemical processes, thereby influencing growth and metabolism in plants during abiotic stress condition^12^. Foliar application of seaweeds shown to ameliorate the effect of abiotic stress with associated changes in several physiological processes, antioxidant defense system, and production of ROS^13^. Auxins, cytokinins, gibberellins, abscisic acid, and brassinosteroids are some phytohormones found in seaweeds^14^. These can act individually or in combinations that contribute to plant growth, development and stress adaptation^15,16^. In the recent gazette notification by the Ministry of Agriculture and Farmers Welfare, the Government of India recognized the importance of biostimulant products as stress alleviating agents and formally approved their inclusion under clause 20C of the Fertilizer Control Order. Inappropriate application of pesticides often triggers a variety of mechanisms viz*.,* inhibition of biological processes such as photosynthesis, cell division, enzyme function, leaf formation or root growth; interference with the synthesis of pigments, proteins or DNA; destruction of cell membranes; alteration in biochemical, physiological^17^ and molecular parameters that ultimately affect the yield and crop quality^18^. All the aforementioned adverse effects are analogous to abiotic stress responses in plants. Hence an attempt was made to investigate the role of red seaweed biostimulants in mitigating fungicidal stress. Research on red seaweed extracts and their formulated products for rice crop is limited in India and mainly done in the plain land rice ecosystem^19,20^. However, the effect of red seaweed on rice concerning fungicidal stress has not been studied. Therefore, in the present study, we aimed to know the biological and molecular effects of two biostimulants (LBD1 and LBS6) obtained from tropical red seaweeds (*Kappaphycus* sp and *Eucheuma* sp) in relieving the fungicidal stress in rice.

## Results

### Evaluation of physiological parameters under fungicidal stress

#### Stomatal behaviour

**Figure 1:**
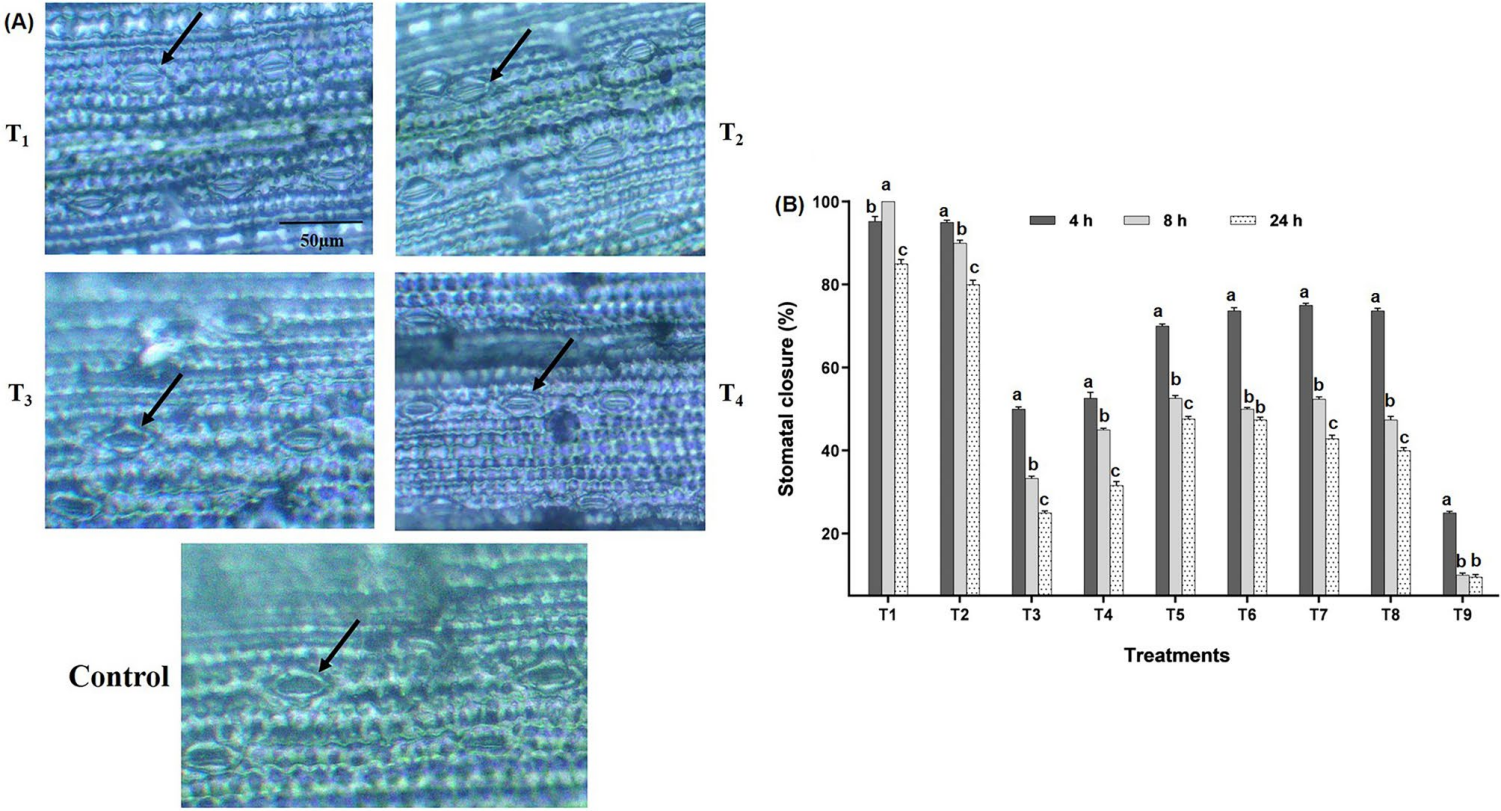
Effect of fungicidal stress on stomatal closure (**A**) stomatal response to different treatments at 4 h after the spray (T_1_: Tricyclazole, T_2_: Carbendazim, T_3_: LBS6 and T_4_: LBD1). (**B**) Bar graph showing the percent stomatal closure in different treatments across the intervals (Legends: T_1_: Tricyclazole, T_2_: Carbendazim, T_3_: LBS6, T_4_: LBD1, T_5_: Tricyclazole + LBS6, T_6_: Tricyclazole + LBD1, T_7_: Carbendazim + LBS6, T_8_: Carbendazim + LBD1, T_9_: Control).

Stomata, the most important physiological structures, were affected by fungicidal stress. Foliar application of tricyclazole and carbendazim increased the stomatal closure by 95% at 4 h after spray (HAS) in the T_1_ and T_2_ (Fig. 1A). However, at 0 h (before the spray), all the stomata were open. At 8 HAS, tricyclazole-treated leaves had a maximum stomatal closure of 100%. Interestingly, foliar application of seaweed biostimulants combined with fungicides decreased stomatal closure by 70 to 75% at 4 HAS (Fig. 1B). However, in tricyclazole and carbendazim-stressed plants, stomatal closure was decreased (40–47.62%) after 24 h of exposure. Plants treated with LBS6 and LBD1 alone witnessed 50, 33.33, 25%, and 52.63, 45, 31.53% stomatal closure at 4, 8, and 24 HAS, respectively. On the other hand, water-sprayed plants showed 25, 10, and 9.52% stomatal closure at 4, 8 and 24 HAS, respectively. In brief, at 0 h all the stomata were open, however after 4 h of fungicide application, the stomatal closure was significantly increased (F = 65.86, *p* < 0.05) and declining trend was recorded at 8 and 24 h when fungicides were combined with seaweed extracts (Supplementary Fig. S1). Hence, the foliar application of fungicides combined with seaweeds significantly decreased stomatal closure.

#### Leaf temperature

**Figure 2:**
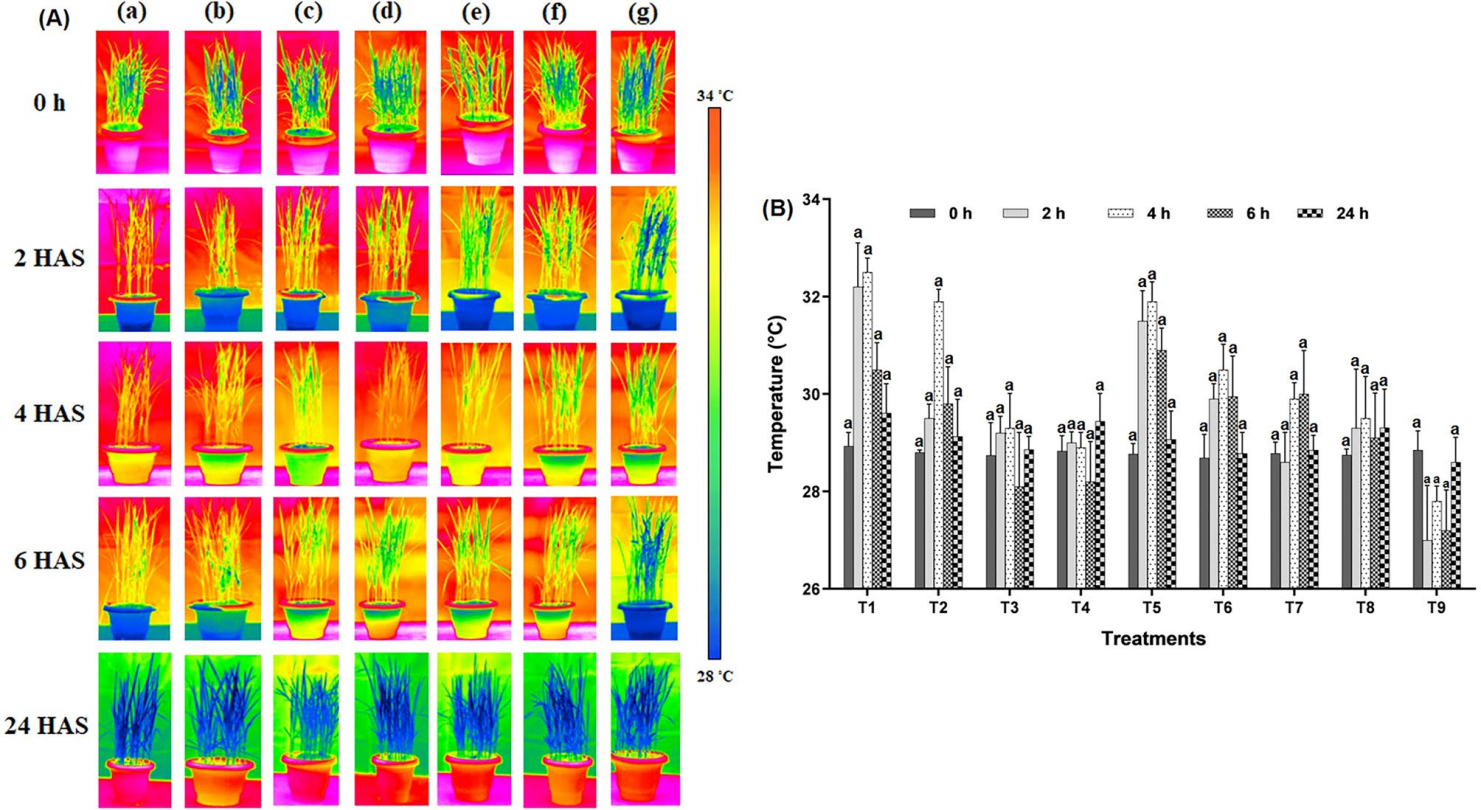
Influence of fungicidal stress on leaf temperature (**A**) Thermographic images of fungicide stressed and control rice plants with different treatments at 0, 2, 4, 6 and 24 HAS. Colour code of measured temperature is included (a) T_1_: tricyclazole, (b) T_2_: carbendazim, (c) T_5_: tricyclazole + LBS6, (d) T_6_: tricyclazole + LBD1, (e) T_7_: carbendazim + LBS6, (f) T_8_: carbendazim + LBD1, (g) T_9_: control. (**B**) Estimation of leaf temperature in different treatments (Legends: T_1_: Tricyclazole, T_2_: Carbendazim, T_3_: LBS6, T_4_: LBD1, T_5_: Tricyclazole + LBS6, T_6_: Tricyclazole + LBD1, T_7_: Carbendazim + LBS6, T_8_: Carbendazim + LBD1, T_9_: Control).

The temperature of fungicide-treated and untreated leaves differed significantly (F = 1.84, *p* < 0.05); the temperature of fungicide-stressed leaves was higher than that of unstressed leaves. The leaves of rice plants displayed typical colours in IR images which were compared to scales given (Fig. 2A). Foliar application of fungicide alone (T_1_ and T_2_) increased the leaf temperature to 32.20, 32.50, 30.50 °C at 2, 4, and 6 HAS. However, in the LBS6 treated plants (T_3_), the temperature increased from 29.20 to 29.30 °C at 2 and 4 HAS, respectively (Fig. 2B). Rice leaves sprayed with LBD1 alone showed a decrease in the temperature over time. While there were no major differences after 24 HAS. In comparison to fungicide treatment alone, the experiment revealed that spraying tricyclazole and seaweed biostimulants together reduced leaf temperature. When tricyclazole was combined with LBS6 and LBD1, the temperature ranged from 28.78 to 31.90 °C at different time intervals than the fungicide alone (29.61 to 32.50 °C). Seaweed biostimulants reduced the temperature by 28.60 (T_7_) and 29.30 (T_8_) in carbendazim-stressed rice plants at 2 HAS. In all the treatments temperature ranged from 28.74 to 28.93 at 0 h, gradually increased at 2 h and trend was peak at 4 HAS. Reduction in the leaf temperature was observed at 6 HAS and after 24 h temperature was normalised (Supplementary Fig. S1).

### Improved biochemical status of rice confers tolerance to fungicidal stress

#### In vivo quantification of reactive oxygen species (ROS)

**Figure 3:**
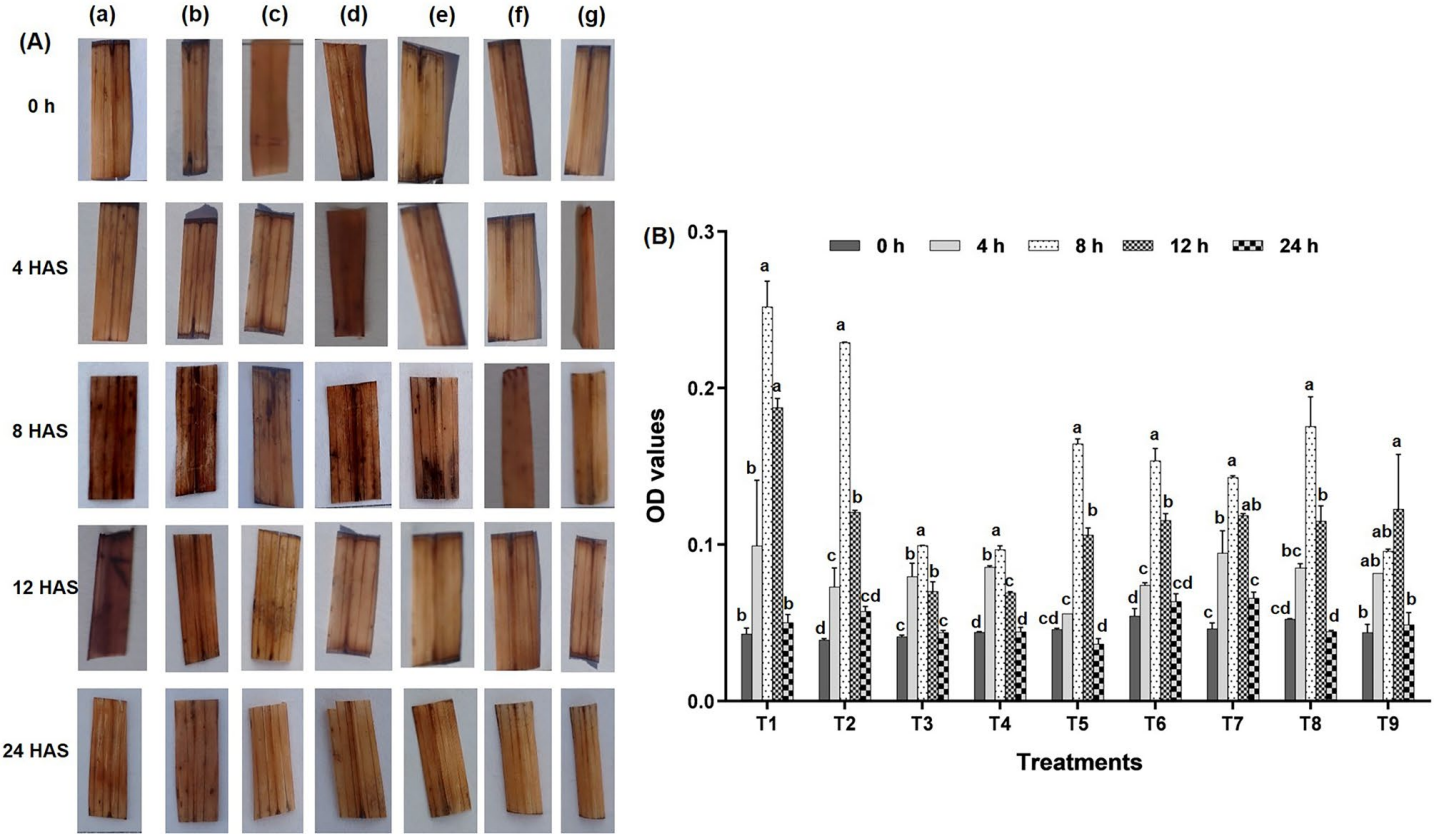
Effect of fungicides on production of hydrogen peroxide (**A**) In vivo localisation of hydrogen peroxide at different interval in response to fungicidal stress (a) T_1_: tricyclazole, (b) T_2_: carbendazim, (c) T_5_: tricyclazole + LBS6, (d) T_6_: tricyclazole + LBD1, (e) T_7_: carbendazim + LBS6, (f) T_8_: carbendazim + LBD1, (g) T_9_: control. (**B**) Estimation of hydrogen peroxide at different intervals (Legends: T_1_: Tricyclazole, T_2_: Carbendazim, T_3_: LBS6, T_4_: LBD1, T_5_: Tricyclazole + LBS6, T_6_: Tricyclazole + LBD1, T_7_: Carbendazim + LBS6, T_8_: Carbendazim + LBD1, T_9_: Control).

**Figure 4:**
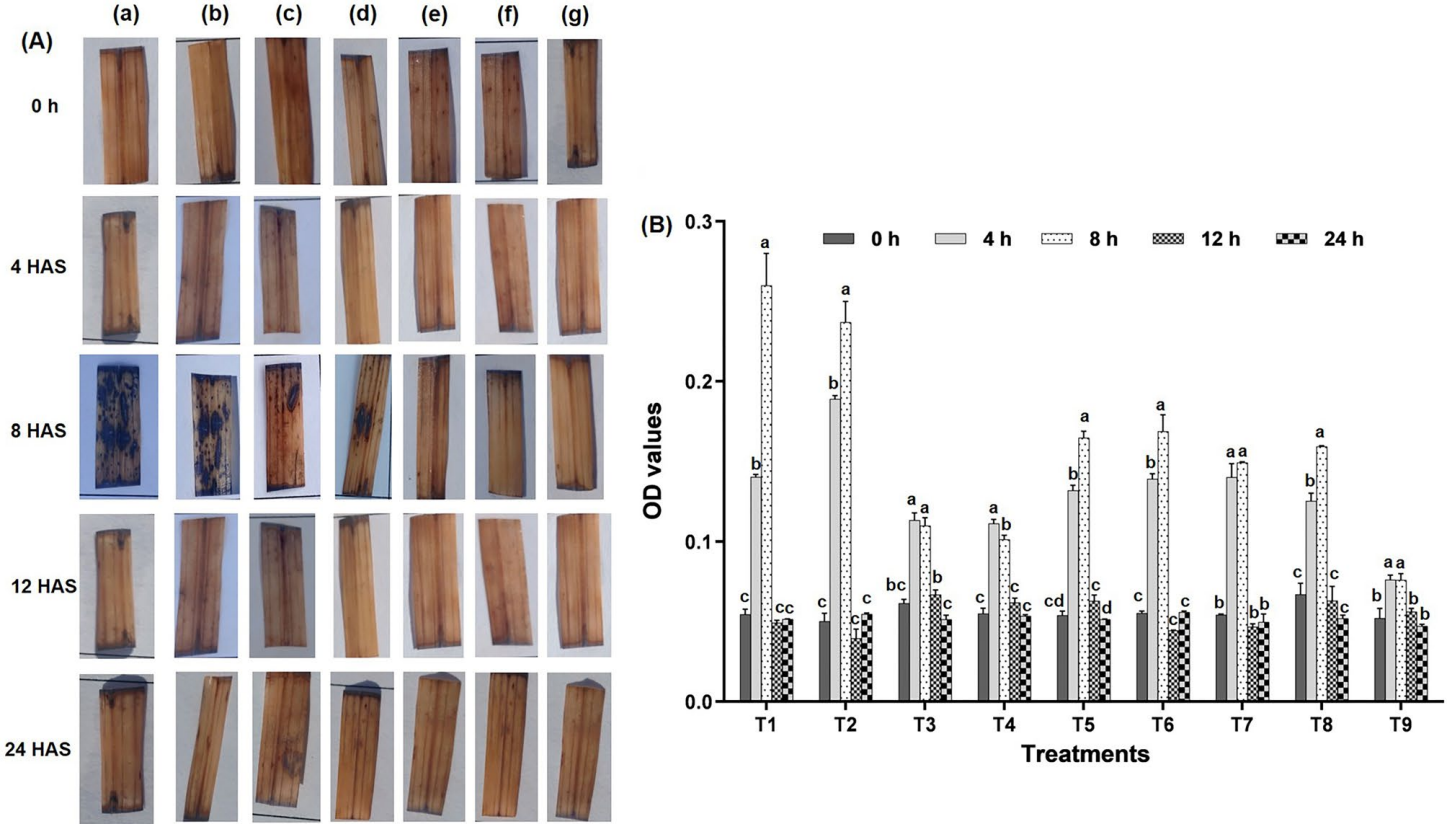
Effect of fungicides on production of super oxide radicals (**A**) In vivo localisation of super oxide radicals at different interval in response to fungicidal stress (a) T_1_: tricyclazole, (b) T_2_: carbendazim, (c) T_5_: tricyclazole + LBS6, (d) T_6_: tricyclazole + LBD1, (e) T_7_: carbendazim + LBS6, (f) T_8_: carbendazim + LBD1, (g) T_9_: control. (**B**) Estimation of super oxide radicals at different intervals (Legends: T_1_: Tricyclazole, T_2_: Carbendazim, T_3_: LBS6, T_4_: LBD1, T_5_: Tricyclazole + LBS6, T_6_: Tricyclazole + LBD1, T_7_: Carbendazim + LBS6, T_8_: Carbendazim + LBD1, T_9_: Control).

In vivo localization of H_2_O_2_ and O_2_^-^ showed no discernible difference in the accumulation of these radicals with biostimulants and water spray, whereas, fungicides and biostimulants exhibited a significant difference. As compared to fungicides, the area and severity of brown spots (an indicator of H_2_O_2_) and blue spots (an indicator of O^2-^) on leaves sprayed with biostimulants (LBS-6 and LBD-1) were less (Figs. 3 and 4).

##### Hydrogen peroxide (H_2_O_2_)

The application of fungicides on rice plants induced stress and resulted in the accumulation of H_2_O_2_. Plants sprayed with tricyclazole (5.85 fold) and carbendazim (5.87 fold) accumulated substantially more H_2_O_2_ than untreated rice plant leaves at 8 HAS. H_2_O_2_ accumulation increased significantly from 0 to 8 HAS, decreased at 12 HAS, but remained significantly higher than 0 and 4 HAS (F = 7.34, p < 0.05), and was normalized at 24 HAS (Fig. 3A and B). There was no distinguishable rise in H_2_O_2_ levels in the sole application of biostimulants and water over different time intervals. There was a significant difference in H_2_O_2_ accumulation with respect to time intervals (0, 8 and 12 HAS) in T_1_ and T_2_ compared to T_3_ and T_4_. A decreasing trend was recorded over the time intervals studied (Supplementary Fig. S2).

##### Superoxide radicals (O_2_^-^)

When O^2-^ were quantified at different spray intervals, foliar application of tricyclazole alone increased O^2-^ accumulation by 2.57 fold at 4 HAS and up to 4.76 fold at 8 HAS compared to 0 h and normalized after 12 HAS (Fig. 4A, B). Application of seaweed bioformulations along with tricyclazole in T_5_ and T_6_ reduced the accumulation of free radicals by 3.06 and 3.05 folds respectively at 8 HAS, which was significantly lower than tricyclazole alone (F = 31.02, *p* < 0.05). In another treatment, carbendazim alone increased free radicals in rice plants by 3.76 times at 4 HAS and up to 4.71 times at 8 HAS. Carbendazim in combination with seaweed biostimulant reduced the free radical accumulation (T_7_ and T_8_) by 2.77 and 2.38 folds, respectively at 8 HAS. There was a significant difference between fungicide alone and combination treatments. The statistical trend was exponential at 8 h and gradually declining trend was recorded at 24 HAS (Supplementary Fig. S2). The use of seaweed biostimulant alone caused stress and free radicals, albeit at a far lower level than fungicides. Compared to fungicides alone, the combination of LBD1 and LBS6 significantly decreased the accumulation of free radicals.

### Seaweed biostimulants on activation of antioxidants

To determine the mechanism of ROS maintenance in seaweed combined fungicidal treatments, we examined various enzymatic antioxidants such as catalase, ascorbate peroxidase, superoxide dismutase, peroxidase and glutathione oxidase to determine their role in combating the excessive ROS production. The trend was exponential in fungicide treatments (T_1_ and T_2_) and peak was recorded at 24 HAS. However, declining trend was observed in combination treatments across the time period (Supplementary Fig. S2).

#### Catalase (CAT)

**Figure 5:**
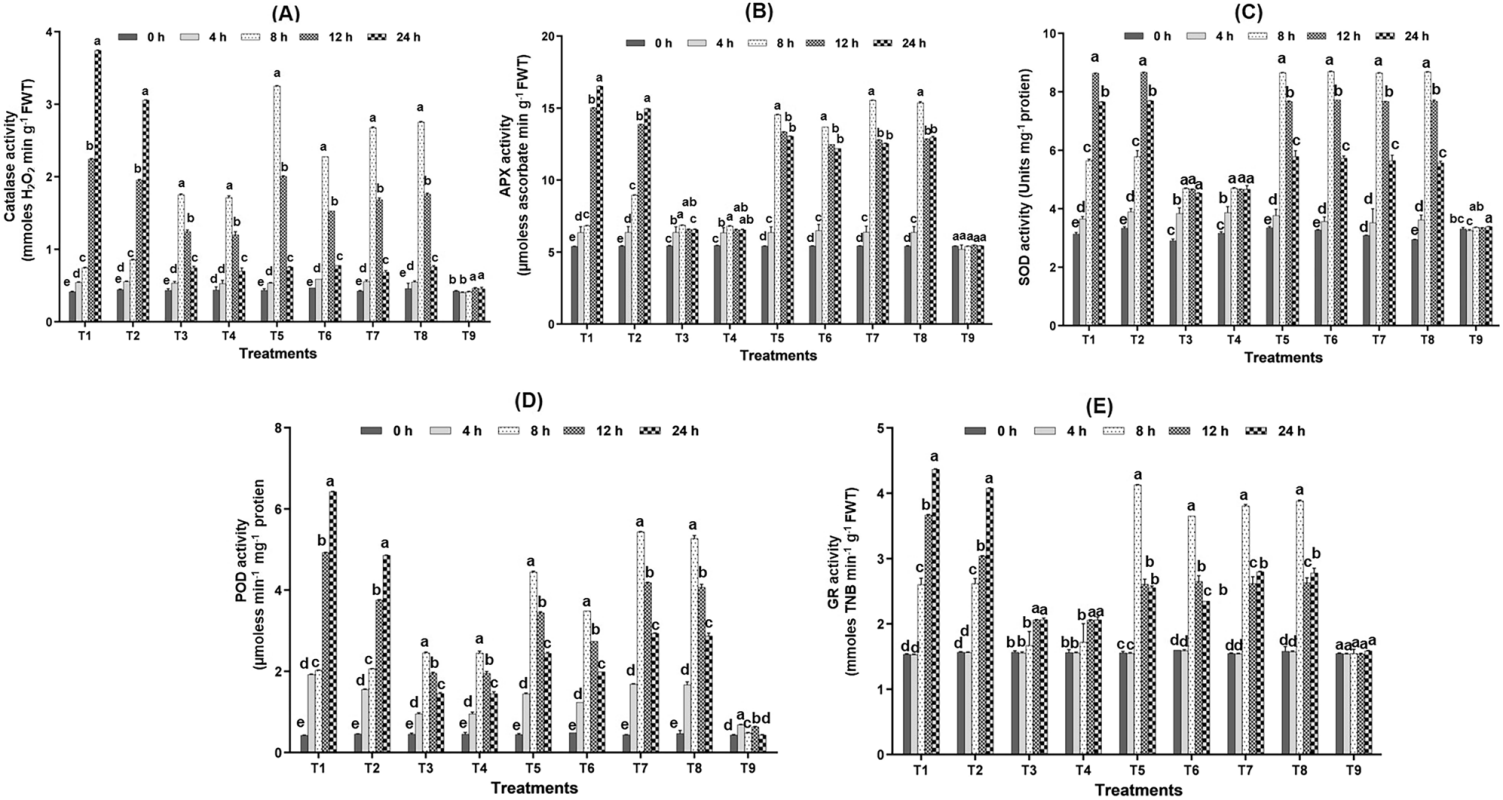
Effect of fungicides and seaweed biostimulants on activity of (**A**) CAT (**B**) APX (**C**) SOD (**D**) POD and (**E**) GR (T_1_: Tricyclazole, T_2_: Carbendazim, T_3_: LBS6, T_4_: LBD1, T_5_: Tricyclazole + LBS6, T_6_: Tricyclazole + LBD1, T_7_: Carbendazim + LBS6, T_8_: Carbendazim + LBD1, T_9_: Control).

Tricyclazole and carbendazim increased catalase activity by 1.30, 1.80, 5.41, 9.20 and 1.25, 1.92, 4.39, 6.87 folds at 4, 8, 12 and 24 HAS respectively (Fig. 5A). Catalase activity was increased by 1.80 and 1.92 fold in the presence of tricyclazole and carbendazim, respectively at 8 HAS, which was lower than seaweed biostimulants. Also, after 24 h, fungicide application alone increased CAT activity. On the other hand, rice leaves treated with fungicides, and seaweed biostimulants showed a decreased CAT activity at 12 and 24 HAS. At 8 HAS, LBS6 and LBD1 alone had a fold shift of 4.03 and 3.90, respectively, which was higher (F = 2677, *p* < 0.05) than the fungicide-treated leaves.

#### Ascorbate peroxidase (APX)

The activity of the APX enzyme was significantly increased in various treatments. Fungicide application alone resulted in increased APX activation even after 24 HAS. In addition, seaweed biostimulants alone did not display much variation in APX activity over time intervals. However, the activity of APX began to increase at 8 HAS in rice leaves treated with fungicides and biostimulants, and it remained stable even after 24 HAS. Tricyclazole and carbendazim increased the APX activity by 1.26 and 1.65 folds at 8 HAS, respectively (Fig. 5B). However, the combined application of fungicide-seaweed biostimulants increased the APX activity from 2.52 to 2.86 fold in different treatments (T_5_–T_8_) at 8 HAS.

#### Superoxide dismutase (SOD)

Fungicide application alone on rice plants increased SOD activity up to 12 h and decreased at 24 HAS. However, seaweed biostimulants alone increased SOD activity at 8 HAS, and it remained stable even after 24 HAS. The rice plants treated with tricyclazole and carbendazim along with seaweed biostimulants (T_5_ and T_7_) intensified the SOD activity by 2.58 and 2.79 folds at 8 HAS. In contrast, SOD activity was reduced in leaves at 12 HAS, evident from Fig. 5C.

#### Peroxidase (POD)

POD activity was increased by foliar application of fungicides at various time intervals, reaching a peak at 24 HAS. Tricyclazole alone increased POD activity from 4.53 to 15.12 fold at 24 HAS (Fig. 5D). However, rice leaves treated with fungicides in combination with seaweed biostimulants significantly increased POD activity at 8 HAS (F = 2550, *p* < 0.0001). At 24 HAS, carbendazim-treated leaves exhibit increased POD activity ranging from 3.40 to 10.63 fold. Carbendazim with seaweed biostimulant, on the other hand, significantly increased POD activity at 8 HAS.

#### Glutathione reductase (GR)

In fungicide-stressed rice plants, GR activity varied with time intervals, with an increasing trend from 8 to 24 h. At 8 HAS, tricyclazole increased GR activity by 1.69 folds; however, at 12 and 24 h, the enzyme activity increased by 2.38 and 2.84 folds, respectively (Fig. 5E). At 24 h, carbendazim-stressed rice plants had increased GR activity from 1.67 to 2.60 folds, slightly lower than tricyclazole-stressed plants. Seaweed bioformulations enhanced the GR activity at 8 HAS when combined with fungicides from 2.30 to 2.65 fold in different treatments (T_5_–T_8_) and declined after 12 h.

### Effect of seaweed biostimulants on an accumulation of plant minerals

Plant minerals, particularly potassium and calcium, are critical in coping with abiotic stress. We also quantified K^+^ and Ca^+^ in different treatments to determine their roles in Stomatal behaviour and ROS formation, respectively.

**Figure 6:**
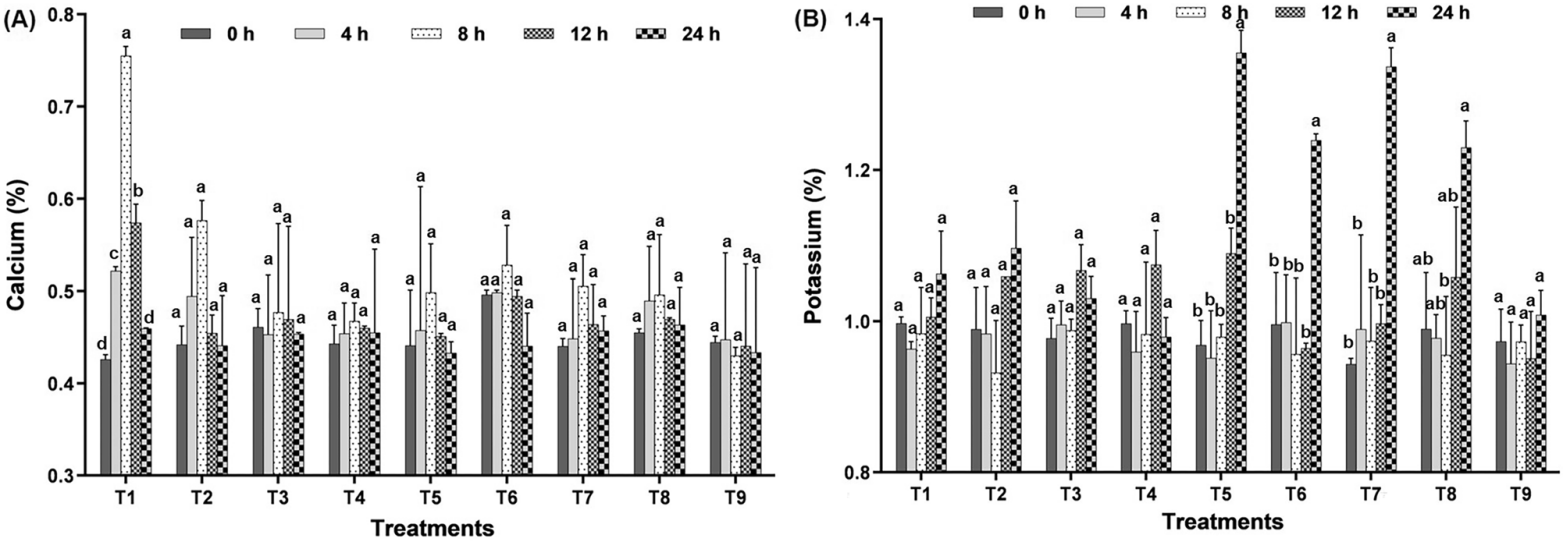
Effect of different treatments on (**A**) calcium and (**B**) potassium content in rice leaves (T_1_: Tricyclazole, T_2_: Carbendazim, T_3_: LBS6, T_4_: LBD1, T_5_: Tricyclazole + LBS6, T_6_: Tricyclazole + LBD1, T_7_: Carbendazim + LBS6, T_8_: Carbendazim + LBD1, T_9_: Control).

The amount of K^+^ ions in rice plants after foliar application of fungicides did not change significantly over time intervals (F = 1.821, *p* < 0.0315). However, after 12 h of spraying, seaweed biostimulant significantly increased the levels of K^+^ ions in fungicide-stressed rice plants (Fig. 6A). There was no significant difference in potassium levels between the two combinations. When rice plants were sprayed with fungicide and seaweed biostimulant, the K^+^ ion level was significantly higher than when rice plants were sprayed with fungicide alone. The trend was exponential in fungicide and combination treatments, however a sudden increase in the potassium level was recorded in T_5_, T_6_ and T_7_ (Supplementary Fig. S1).

When compared to water control, applying fungicide alone to rice plants increased the Ca^+^ content. Except for the tricyclazole stress (F = 1.82, *p* < 0.05), there was no significant difference in the Ca^+^ quality of the leaves across the treatments at various intervals. The Ca^+^ content was substantially reduced by foliar application of fungicide combined with seaweed biostimulant. At 8 HAS tricyclazole alone increased the Ca^+^ content to 0.76%; but, when LBS6 and LBD1 were combined with tricyclazole, the Ca^+^ content was reduced by 0.50 and 0.53%, respectively (Fig. 6B).

### Enhanced expression of stress-responsive genes

**Figure 7:**
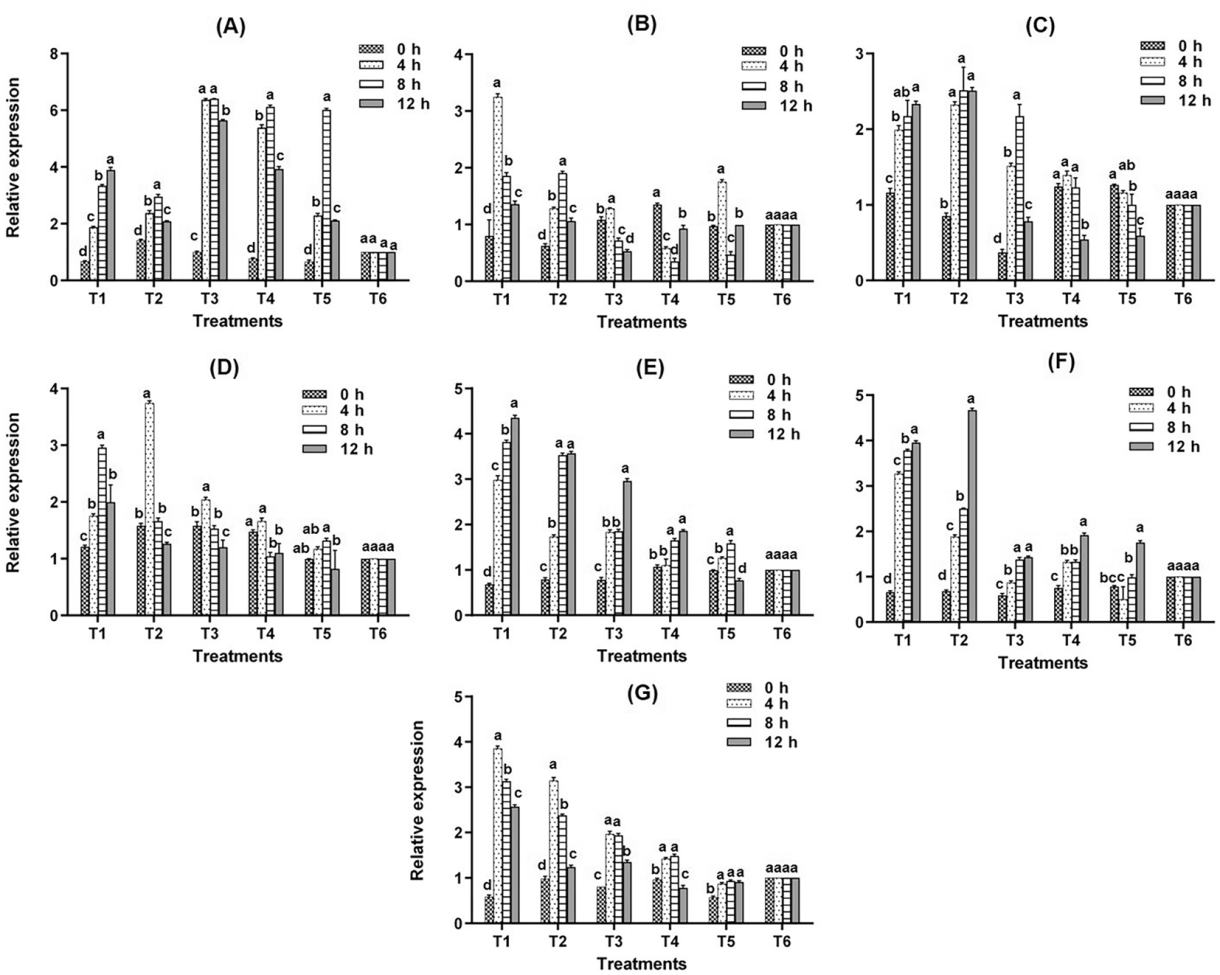
Response of (**A**) *APX,* (**B**) *E2F,* (**C**) *HSFA2A,* (**D**) *HSFB2B,* (**E**) *HSFB4C,* (**F**) *HSFC1A* and (**G**) *OsZIP-12* to fungicidal stress in different treatments (T_1_: Tricyclazole, T_2_:Carbendazim, T_3_:Tricyclazole + LBS6, T_4_:Carbendazim + LBS6 and T_5_:LBS6, T_6_: Water Control).

Transcript analysis of APX, a ROS scavenging gene, E2F, elongation factor, Heat Shock Factors HSFA2A, HSFB2B, HSFB4C, HSFC1A, and ZIP12 at 0, 4, 8, and 12 h of different treatments were carried out to determine the molecular mechanism of seaweed biostimulants in relieving fungicide stress (Fig. 7A–G). Regression analysis showed gradual increase in stress responsive transcripts at 4 and 12 HAS across the treatments. Declining trend was observed at 12 h with respect to APX, HSFA2A, HSFB2B and ZIP12 (Supplementary Fig. S3).

Foliar application of fungicides combined with seaweed biostimulants (T_3_ and T_4_) resulted in a slightly observable induction of *APX* at 4 h of stress with a fold shift of 6.36, which steadily decreased at 12 h, indicating the existence of a strong defense mechanism. However, *APX* in the fungicide-treated leaves decreased after 8 h (Fig. 7A). Fungicide application increased the transcript level of *E2F* by 3.24 and 1.28 folds, respectively, in tricyclazole and carbendazim stressed rice leaves at 4 HAS (Fig. 7B). However, when the same fungicide concentration was sprayed alongside LBS-6, the expression level of *E2F* was reduced to 1.28 and 0.58, respectively (T_3_ and T_4_). The level of *E2F* steadily decreased at 8 and 12 h, suggesting short-term stress (Fig. 7B). The response of *HSF* (Heat shock factors) transcripts was studied over time. At various points in time, HSFA2A, HSFB2B, *HSFB4C*, and *HSFC1A* were upregulated. At 8 h after tricyclazole and carbendazim sprays, *HSFA2A* and *HSFB4C* expression levels were high, with 2.17, 2.15, and 3.82, 3.53, respectively (Fig. 7C, E). At 12 h, the transcript level of *HSFC1A* increased significantly, with a relative expression of 3.94 and 4.66 in tricyclazole and carbendazim stressed plants, respectively (Fig. 7F). When combined with fungicides, LBS6 significantly decreased the expression level of *HSFC1A* by 1.43, 1.92. *ZIP12* transcript was an early responsive gene in fungicide stressed plants, with a fold increase of 3.85 and 3.15 at 4 h and then decreasing at 8 and 12 h (Fig. 7G). However, the expression level was less in case of combination treatments of both the fungicides, which is evidence for the stress-relieving capacity of seaweed bioformulation.

## Discussion

Plants, being sessile, are relentlessly challenged by various environmental stresses that limit their growth and productivity^21,22^. Due to the complex metabolic pathways involved in stress tolerance, limited success has been achieved in generating stress-tolerant crops through genetic engineering^21,23^. Another sustainable approach to improve stress tolerance in plants is seaweed biostimulants^24^. Abiotic stresses such as fungicidal stress largely influence plant development. To cope with stress, plants initiate several molecular, cellular and physiological changes to respond and adapt to such stresses^25^. In this study, the effect of fungicide application in inducing the abiotic-like stress in the rice plant was studied. An attempt was made to analyse the effects of two seaweed biostimulants in alleviating the fungicide induced stress. The results from the stomatal aperture study indicated that the fungicide application led to the higher stomatal closure. However, it was drastically reduced when fungicides were applied in combination with the seaweed biostimulants. This effect is attributed to many phytohormones in the seaweeds *i.e.,* gibberellic acid and cytokinins, which influence the stomatal opening during the fungicidal stressed condition as reported previously for other abiotic stresses^26^. Seaweed biostimulants were reported as capable of maintaining a strong stomatal control during the phase of abiotic stress^27^.

Since higher temperature is negatively correlated to plant vitality^28^, IR thermography was used to establish differences in the plant stress in different treatments represented as the surface temperature of leaves. A direct correlation was reported between leaf temperature and stress^29^. There was an increase in leaf temperature when plants were treated with fungicides alone. Seaweed biostimulants acted as anti-stress agents in reducing the leaf temperature across the period studied. Abiotic stress reduces transpirational cooling, therefore increasing leaf temperature^30^. In the previous studies, seaweed extract from *Ascophyllum nodosum* was shown to help soybean plants withstand severe drought conditions by regulating leaf temperature, turgor, and several stress-responsive genes^31,32^.

Plants under stress conditions tend to produce ROS that includes superoxide and hydroxyl radicals^33–35^. In the present study, ROS quantification suggested that fungicide application led to the higher production of ROS mimicking the abiotic stress like condition in the plant. Seaweed biostimulants alleviate the excessively produced ROS by inducing antioxidants defense during stress conditions^4^. Therefore, we tested different combinations of seaweed extracts and fungicides to study the suppression of ROS production. We estimated the different enzymatic antioxidants activity, which suggested that seaweed biostimulants used in the study effectively alleviated the stress induced by fungicides in terms of reducing ROS production. In a nutshell, superoxide radicals were recorded to be higher at 4 h after the spray, whereas H_2_O_2_ was at its peak at 8 h (T_1_ and T_2_). When the enzymatic antioxidants were considered, CAT, APX and SOD were drastically increased at 8 h. However, POD and GR activities were more at 12 h and 24 h of spray respectively. The dismutation of superoxide radicals into H_2_O_2_ and oxygen protects the cells from various stresses and is catalyzed by SOD. Hence at 8 h after the spray, H_2_O_2_ was higher, which was dismuted from O_2_^-^ through SOD. ROS was stabilized at 12 h due to the activity of POD, APX, CAT and GR. The present study shows that seaweed biostimulant (LBS6) reduced the stress induced by fungicides and normalized the cells within 8 h of the spray. It has been reported previously that plants have their own innate defense mechanism to cope with the stresses. The application of seaweed biostimulant will increase the efficiency of the plants to activate such defense rapidly^36^. The effect of seaweed extracts was studied on many crops during abiotic stress conditions and shown to reduce the accumulations of ROS, which was attributed to enhanced activity of enzymatic antioxidants such as SOD, CAT, and APX^10,37–39^.

Ionic (K^+^ and Ca^2+^) imbalance is a major abiotic stress consequence. Calcium signalling pathways interact with other cellular signalling systems suchs as ROS and leads to the production of ROS during stress^40^. Increased Ca^2+^ levels activated ROS-generating enzymes and the development of free radicals as a stress-related defense mechanism^41^. The present study showcased that, calcium plays a crucial role in the production of ROS during fungicidal stress. Potassium plays an essential role in enzyme activation, osmoregulation, stomatal movement, energy transfer, cation–anion balance and stress resistance^42^. In the current study, potassium concentration elevated at 12 and 24 HAS with fungicide and biostimulant resulting in stomatal opening. In the previous studies, a higher K^+^ level has been reported to alter the activity of SOD, CAT, POD against H_2_O_2_ production during oxidative stress^10,41^. The application of *A. nodosum*-based extracts reportedly alleviated the salinity stress by improving nutrient uptake (Ca^2+^ and K^+^) in avocado plants^43^. This report, perhaps correlated to the present study, which showed the role of seaweed biostimulants in alleviating fungicidal stress.

**Figure 8:**
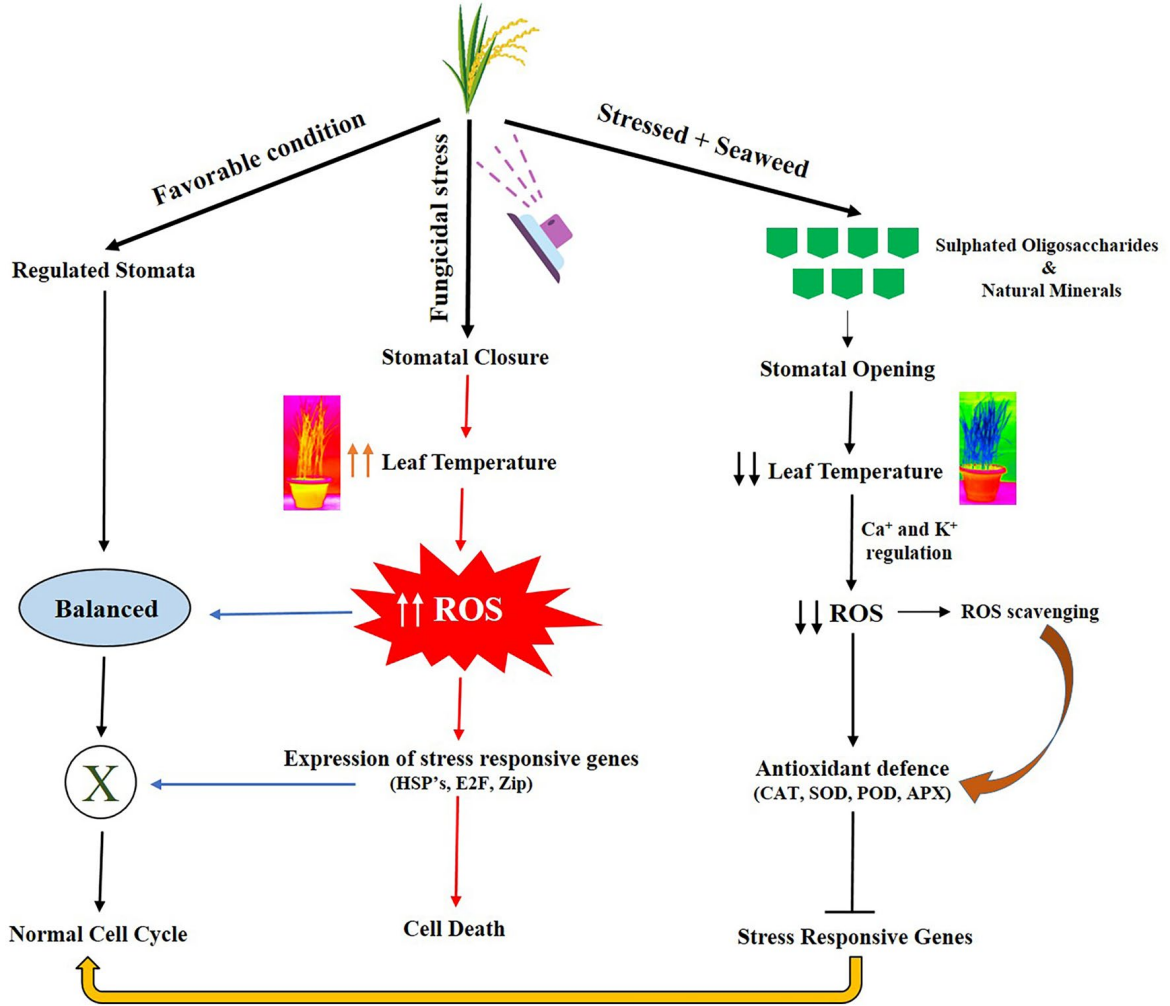
Schematic representation depicting the mechanism of red seaweed biostimulants in imparting fungicidal stress tolerance.

Expression of stress-sensitive genes was less in fungicide + seaweed treatments (T_3_ and T_4_) compared to fungicides alone (T_1_ and T_2_), which provides evidence for the stress-relieving capacity of seaweed biostimulants. Many reports indicated the role of seaweeds in abiotic stress management, but none of the reports describe fungicidal stress, and therefore, this is the first report on fungicidal stress focusing on gene expression studies. The application of seaweed extracts significantly influenced the expression of genes involved in the biosynthesis and transport of flavonoids, which protect plants from ROS-induced oxidative damage during stress^4^. qPCR analysis showed enhanced expression of stress-responsive wheat MAP kinase, WRKY transcription factor, and antioxidative genes with seaweed treatment during stress^10^. From the present study, it can be summarized that the seaweed biostimulants activity is due to the coordination of molecular and biochemical changes which improved better physiology, ROS scavenging, ionic balance and normalization of plants under fungicidal stress (Fig. 8).

## Materials and methods

### Sources of biostimulants and fungicides

The seaweed biostimulants (LBD-1 and LBS-6, 20% solid extract) used in the study were provided by Sea6Energy Pvt Ltd, Bengaluru, India. Briefly, the tropically grown red seaweed biomass of *Kappaphycus* sp and *Eucheuma* sp were processed by following two patented technologies (US10358391B2 and PCT/IN2019/050831) to obtain solid and liquid fractions. LBS-6 product was prepared by blending the extracts of liquid and solid fractions while LBD-1 was prepared from the extracts of the solid fraction. The liquid extracts are rich in natural minerals present in the seaweed while the solid fractions are rich in bioactive sulphated galacto oligosaccharides^44,45^. The total Sodium (% w/w) content of LBS6 and LBD1 is 1.18 ± 0.13 and 0.51 ± 0.05 respectively. It was estimated by ICP-OES (Inductively Coupled Plasma Optical Emission spectroscopy) technique. Commonly used fungicides viz*.,* tricyclazole 75%WP (Corteva Agriscience) and carbendazim 50% WP (BASF, India) were used in the study.

### Plant growth and stress induction in the glasshouse

In this study, we have used a cultivated popular rice variety *i.e.,* cv. IR-64 which was grown under glasshouse conditions at the Glass House Facility, Department of Plant Pathology, University of Agricultural Sciences (UASB), Gandhi Krishi Vignan Kendra (GKVK), Bangalore, India. The glasshouse studies and experimental research including the collection of plant material, complied with institutional, national and international guidelines and legislation. This plant species has not been listed as endangered and the collection sites of this study were not inside the national park or other restricted forest areas. The experiments were carried out in controlled conditions. Thirty days old plants were subjected to different treatment viz., T_1_ : tricyclazole 75%WP (0.6 g L^-1^), T_2_ : carbendazim 50%WP (1.0 g L^-1^), T_3_ : LBS6 20% (1 mL L^-1^), T_4_ : LBD1 20% (2 mL L^-1^), T_5_ : tricyclazole 75%WP + LBS6 20% (0.6 g + 1.0 mL L^-1^), T_6_ : tricyclazole 75%WP + LBD1 20% (0.4 g + 2 mL L^-1^), T_7_: carbendazim 50%WP + LBS6 20% (1.0 g + 1 mL L^-1^), T_8_ : carbendazim 50%WP + LBD1 20% (0.6 g + 2 mL L^-1^), T_9_: untreated check (Water spray). All the treatments in the experiment were replicated twice. A total of fifteen plants in each replications were sprayed with 250 mL of mixture until the plants were completely drenched.

### Analysis of physiological parameters

At various time intervals, physiological parameters related to abiotic stress, such as stomatal behaviour and leaf temperature, were examined. Stomatal behaviour was determined by counting closed and opened stomata using the xylene impression method at 0 (before spray), 4, 8, and 24 HAS, and the percentage of stomatal closure was calculated. At 0, 2, 4, 6, and 24 h, digital thermographic images of the leaf were taken. The temperature of the leaf was measured using a thermal imager (VARIOCAM, Jenoptik-Germany) and analysed with the software package IRBIS 3 (Infratec, Germany).

### Analysis of biochemical parameters

Fresh leaf samples were collected from stressed and unstressed (control) rice plants at 0, 4, 8, 12, and 24 HAS to assess the levels of ROS, antioxidants, and plant minerals.

#### In vivo quantification of Reactive Oxygen Species (ROS)

##### Determination of hydrogen peroxide (H_2_O_2_) by diaminobenzidine (DAB) Staining

To quantify H_2_O_2_, freshly collected leaves were immersed in a staining solution containing 1 mg mL^-1^ DAB solution at pH 3.8. The development of colour due to H_2_O_2_ was determined according to Ramanjulu^46^ protocol, accompanied by the photography of stained leaves.

##### Determination of superoxide anion (O_2_^-^) radicals by histochemical detection technique

Collected leaf samples were immersed with the abaxial side up in 100 mL of staining solution containing 0.1% (w/v) Nitro Blue Tetrazolium (NBT), 10 mM sodium azide, and 50 mM potassium phosphate at pH 6.4. The leaves were vacuum infiltrated (∼100– 150 mbar for 1 min) and then released gently. The same procedure was repeated 2–3 times until the leaves were completely infiltrated. Then the leaves were incubated in 10 mL of staining solution (0.1% NBT) for 15 min followed by cool fluorescent light for 20 min. The reaction was stopped with 95% ethanol. Chlorophyll was removed by a succession of washes with fresh ethanol. Superoxide ions reacted with NBT and appeared as a blue stain. The stained leaves were photographed and superoxide radicals were quantified according to the protocol described by^46^.

#### Estimation of antioxidants

About 200 mg of leaf tissue was ground into a fine powder using liquid nitrogen to estimate antioxidants. Every powdered sample was precisely weighed before being thoroughly homogenized in 1.2 mL of 0.2 M potassium phosphate buffer (pH 7.8 with 0.1 mM Ethylene Diamine Tetra Acetic acid). The homogenized samples were centrifuged at 15,000 × g for 20 min at 4 °C, the supernatant was discarded, and the pellet was resuspended in 0.8 mL of potassium phosphate buffer. The suspension was centrifuged for another 15 min at 15,000 × g at 4 °C, and the supernatant was collected. The combined supernatants were kept on ice and were used to test the activities of various antioxidant enzymes. Catalase (CAT: EC 1.11.1.6) and ascorbate peroxidase (APX: EC 1.11.1.11) were measured using the Aebi^47^ method and a modified ^48^ method, respectively. Superoxide dismutase (SOD: EC 1.15.1.1), peroxidase (POD: EC 1.11.1.7) and glutathione reductase (GR: EC 1.6.4.2) were calculated as defined by Rao et al.^49^.

#### Estimation of plant minerals

Collected leaf samples were dried for digestion in an oven at 70 °C for 48 h to assess plant minerals (calcium and potassium). One gram of dried and ground leaf samples were digested in 25 mL of the wet di-acid mixture (Di-acid mixture was prepared by mixing nine parts of HNO_3_ with four parts of HClO_4_). After moistening the leaf sample with the mixture, it was put in an electric sand bath. The contents were heated to 180–200 °C before white fumes appeared and the solution became colorless. When the flask contents were dried or yellowish at the end of digestion, 5 mL of the di-acid mixture were added. Twenty millilitres of distilled water was added and thoroughly stirred before being filtered through filter paper (Whatman No. 1) into a volumetric flask and filled up to 100 mL with distilled water. The potassium and calcium content of leaf samples was calculated using the methods defined by Jackson^50^ (Flame photometry) and Baruah^51^, respectively.

### Molecular changes induced by the seaweed biostimulants in response to fungicidal stress

#### RNA extraction and cDNA synthesis

RNeasy plant mini kit (QIAGEN, Germany) was used to extract total RNA from rice leaves, which was then converted to cDNA using the PrimeScript™ RT reagent Kit (Takara, Japan) according to the manufacturer's instructions.

#### Quantitative real-time PCR analysis

The transcript abundance of *APX, E2F, HSFA2A, HSFB2B, HSFB4C, HSFC1A* and *ZIP12* genes was studied in the leaf tissue of selected treatments viz*.,* T_1_ : tricyclazole 75%WP (0.6 g/L), T_2_ : carbendazim 50%WP (1.0 g/L), T_3_ : tricyclazole 75%WP + LBS6 (0.4 g + 1.0 mL/L), T_4_ : carbendazim 50%WP + LBS6 (1.0 g + 1 mL /L), T_5_ : LBS6 (1 mL/L), T_6_ : control (water spray). The effective treatments from other physiological and biochemical parameters were selected and subjected to the analysis. Leaf samples were collected at 0, 4, 8 and 12 HAS. Real-time PCR amplifications were performed using SYBR Green (Takara) with gene-specific primers, and rice specific ubiquitin gene was used as a reference gene. Relative expression was calculated using the comparative cycle threshold method described by Livak and Schmittgen^52^.

### Statistical analysis

The mean values, standard deviations, and standard error were determined for each experiment after replicating it twice. To assess the importance of the discrepancy between the means of regulation and various stress treatments, two-way analysis of variance was conducted using GraphPad Prism 5 software and Fisher's least significant difference (LSD) at *P* ≤ 0.05. The data shown in figures are means of replicates and error bars are based on standard deviation (SD). Duncan’s Multiple Range Test (DMRT) and quadratic regression analysis was carried out. Graphical representation of the regression analysis is furnished in supplementary information.

## Conclusion

Natural, sustainable, or environmentally friendly agriculture growth approaches are becoming increasingly popular. Modern agriculture aims to reduce fungicidal use and the antagonistic effects of fungicides while maintaining production and quality. The current findings show that seaweed biostimulants could relieve abiotic stress via physiological, biochemical, and molecular mechanisms. When seaweed biostimulants were applied to rice plants, it was found that all of the physio-biochemical parameters that were triggered worked synergistically to increase plant health under stress conditions. Overall, the fungicide-induced ROS production may have been minimized by the sulphated oligosaccharides present in the seaweed biostimulants. This was evident in the seaweed treated plants which showed reduced ROS accumulation, increased antioxidants, reduced leaf temperature and expression of stress related genes. The possible mechanism underpins the stimulation of stress-responsive genes and Transcription factors (TF’s) for regulating various physiological and biochemical pathways, leading to fungicidal stress. Hence, these seaweed biostimulants can be included in organic farming since they reduce pesticide use and maintain plant health.

## Supplementary Information

Supplementary Information 1.

Supplementary Information 2.

Supplementary Information 3.

Supplementary Information 4.

## References

[1] Ansari MUR, Shaheen T, Bukhari SA, Husnain T (2015). Genetic improvement of rice for biotic and abiotic stress tolerance. Turk. J. Bot..

[2] Van Oosten MJ, Pepe O, De Pascale S, Silletti S, Maggio A (2017). The role of biostimulants and bioeffectors as alleviators of abiotic stress in crop plants. Chem. Biol. Technol. Agric..

[3] Pal A, Dwivedi SK, Maurya PK, Kanwar P (2015). Effect of seaweed saps on growth, yield, nutrient uptake and economic improvement of maize (sweet corn). J. Appl. Nat. Sci..

[4] Jithesh MN, Shukla PS, Kant P, Joshi J, Critchley AT, Prithiviraj B (2019). Physiological and transcriptomics analyses reveal that *Ascophyllum nodosum* extracts induce salinity tolerance in arabidopsis by regulating the expression of stress responsive genes. J. Plant Growth Regul..

[5] Dresselhaus T, Hückelhoven R (2018). Biotic and abiotic stress responses in crop plants. Agronomy.

[6] Lin PA, Chen Y, Ponce G, Acevedo FE, Lynch JP, Anderson CT, Ali JG, Felton GW (2022). Stomata-mediated interactions between plants, herbivores, and the environment. Trends Plant Sci..

[7] Battacharyya D, Babgohari MZ, Rathor P, Prithiviraj B (2015). Seaweed extracts as biostimulants in horticulture. Sci. Hortic..

[8] Blunden G, Morse PF, Mathe I, Hohmann J, Critchley AT, Morrell S (2010). Betaine yields from marine algal species utilized in the preparation of seaweed extracts used in agriculture. Nat. Prod. Commun..

[9] Audibert L, Fauchon M, Blanc N, Hauchard D, Ar Gall E (2010). Phenolic compounds in the brown seaweed Ascophyllum nodosum: distribution and radical-scavenging activities. Phytochem. Anal..

[10] Patel K, Agarwal P, Agarwal PK (2018). *Kappaphycus alvarezii* sap mitigates abiotic-induced stress in Triticum durum by modulating metabolic coordination and improves growth and yield. J. Appl. Phycol..

[11] Reynolds-Henne CE, Langenegger A, Mani J, Schenk N, Zumsteg A, Feller U (2010). Interactions between temperature, drought and stomatal opening in legumes. Environ. Exp. Bot..

[12] Pathak, J., Ahmed, H., Kumari, N., Pandey, A. Role of Calcium and Potassium in Amelioration of Environmental Stress in Plants. *Prot. Chem. Agents Amelior. Plant Abiotic Stress* **13**, 535–562 (2020)

[13] Trivedi K, Vijay Anand KG, Vaghela P, Ghosh A (2018). Differential growth, yield and biochemical responses of maize to the exogenous application of Kappaphycus alvarezii seaweed extract, at grain-filling stage under normal and drought conditions. Algal Res..

[14] Stirk WA, Tarkowská D, Turečová V, Strnad M, van Staden J (2014). Abscisic acid, gibberellins and brassinosteroids in Kelpak®, a commercial seaweed extract made from Ecklonia maxima. J. Appl. Phycol..

[15] Rouphael Y, De Micco V, Arena C, Raimondi G, Colla G, De Pascale S (2017). Effect of Ecklonia maxima seaweed extract on yield, mineral composition, gas exchange, and leaf anatomy of zucchini squash grown under saline conditions. J. Appl. Phycol..

[16] Ottaiano, L., Mola, I. Di, Cozzolino, E., El-nakhel, C. & Rouphael, Y. Biostimulant application under different nitrogen fertilization levels : Assessment of yield, leaf quality, and nitrogen metabolism of tunnel-grown lettuce. *Agronomy*, **11**, 1–13 (2021).

[17] Parween T, Jan S, Mahmooduzzafar S, Fatma T, Siddiqui ZH (2016). Selective effect of pesticides on plant—a Review. Crit. Rev. Food Sci. Nutr..

[18] Liu N, Zhu L (2020). Metabolomic and transcriptomic investigation of metabolic perturbations in Oryza sativa L. Triggered by Three Pesticides. Environ. Sci. Technol..

[19] Devi NL, Mani S (2015). Effect of seaweed saps *Kappaphycus alvarezii* and *Gracilaria* on growth, yield and quality of rice. Indian J. Sci. Technol..

[20] Sahana BN, Prasanna Kumar MK, Mahesh HB, Buela Parivallal P, Puneeth ME, Gautam C, Girish TR, Nori S, Suryanarayan S (2022). Biostimulants derived from red seaweed stimulate the plant defence mechanism in rice against Magnaporthe oryzae. J. Appl. Phycol..

[21] Agarwal PK, Shukla PS, Gupta K, Jha B (2013). Bioengineering for salinity tolerance in plants: State of the art. Mol. Biotechnol..

[22] Shukla PS, Borza T, Critchley AT, Prithiviraj B (2016). Carrageenans from red seaweeds as promoters of growth and elicitors of defense response in plants. Front. Mar. Sci..

[23] Mickelbart MV, Hasegawa PM, Bailey-Serres J (2015). Genetic mechanisms of abiotic stress tolerance that translate to crop yield stability. Nat. Rev. Genet..

[24] Shukla PS (2019). Ascophyllum nodosum-based biostimulants: Sustainable applications in agriculture for the stimulation of plant growth, stress tolerance, and disease management. Front. Plant Sci..

[25] Wu L (2013). Assessment of shifts in microbial community structure and catabolic diversity in response to Rehmannia glutinosa monoculture. Appl. Soil Ecol..

[26] Santakumari M, Fletcher RA (1987). Reversal of triazole-induced stomatal closure by gibberellic acid and cytokinins in Commelina benghalensis. Physiol. Plant..

[27] Santaniello A, Scartazza A, Gresta F, Loreti E, Biasone A, Di Tommaso D, Piaggesi A, Perata P (2017). Ascophyllum nodosum seaweed extract alleviates drought stress in Arabidopsis by affecting photosynthetic performance and related gene expression. Front. Plant Sci..

[28] Narayanan S (2018). Effects of high temperature stress and traits associated with tolerance in wheat. Open Access J. Sci..

[29] Oerke EC, Steiner U, Dehne HW, Lindenthal M (2006). Thermal imaging of cucumber leaves affected by downy mildew and environmental conditions. J. Exp. Bot..

[30] Yordanov VV, Tsonev T (2000). Plants responses to drought, acclimation, and stress tolerence. Photosynthetica.

[31] Martynenko A, Shotton K, Astatkie T, Petrash G, Fowler C, Neily W, Critchley AT (2016). Thermal imaging of soybean response to drought stress: the effect of Ascophyllum nodosum seaweed extract. Springerplus.

[32] Shukla PS, Borza T, Critchley AT, Hiltz D, Norrie J, Prithiviraj B (2018). Ascophyllum nodosum extract mitigates salinity stress in Arabidopsis thaliana by modulating the expression of miRNA involved in stress tolerance and nutrient acquisition. PLoS One.

[33] Mittler R (2002). Oxidative stress, antioxidants and stress tolerance. Trends Plant Sci..

[34] Farooq M, Wahid A, Kobayashi N, Fujita D, Basra SMA (2009). Review article Plant drought stress : effects, mechanisms and management. Agron. Sustain. Dev.

[35] Shivakumara TN, Sreevathsa R, Dash PK, Sheshshayee MS, Papolu PK, Rao U, Tuteja N, UdayaKumar M (2017). Overexpression of Pea DNA Helicase 45 (PDH45) imparts tolerance to multiple abiotic stresses in chili (Capsicum annuum L.). Sci. Rep..

[36] Fan D, Hodges DM, Critchley AT, Prithiviraj B (2013). A commercial extract of brown macroalga (Ascophyllum nodosum) affects yield and the nutritional quality of spinach In Vitro. Commun. Soil Sci. Plant Anal..

[37] Mansori M, Chernane H, Latique S, Benaliat A, Hsissou D, El Kaoua M (2015). Seaweed extract effect on water deficit and antioxidative mechanisms in bean plants (Phaseolus vulgaris L.). J. Appl. Phycol..

[38] Elansary HO, Yessoufou K, Abdel-Hamid AM, El-Esawi MA, Ali HM, Elshikh MS (2017). Seaweed extracts enhance salam turfgrass performance during prolonged irrigation intervals and saline shock. Front. Plant Sci..

[39] Carvalho MEA, De Camargo E Castro PR, Gaziola SA, Azevedo RA (2018). Is seaweed extract an elicitor compound? Changing proline content in drought-stressed bean plants. Comun. Sci..

[40] Steinhorst L, Kudla J (2013). Calcium and reactive oxygen species rule the waves of signaling. Plant Physiol..

[41] Ahmad P, Abdel Latef AA, Abd Allah EF, Hashem A, Sarwat M, Anjum NA, Gucel S (2016). Calcium and potassium supplementation enhanced growth, osmolyte secondary metabolite production, and enzymatic antioxidant machinery in cadmium-exposed chickpea (Cicer arietinum L.). Front. Plant Sci..

[42] Wang M, Zheng Q, Shen Q, Guo S (2013). The critical role of potassium in plant stress response. Int. J. Mol. Sci..

[43] Bonomelli C, Celis V, Lombardi G, Mártiz J (2018). Salt stress effects on avocado (persea americana mill) plants with and without seaweed extract (ascophyllum nodosum) application. Agronomy.

[44] Nori, S.S., Kumar, S., Khandelwal, S., Suryanarayan, S. Biostimulant formulation for improving plant growth and uses thereof. PCT, (2017), US10358391B2.

[45] Girish, T., Vantharam, V., Malhotra, P., Bhose, S.P., Sekar, N. et al. Composition comprising sulphated galactose, and implementations thereof. (2020), PCT/IN2019/050831.

[46] Walker, J. M. & Sunkar, R. *Plant Stress Tolerance - Methods and Protocols*. *Methods in Molecular Biology. ***639**, 294–295 (2010).

[47] Aebi H (1984). [13] Catalase in Vitro. Methods Enzymol..

[48] Nakano Y, Asada K (1981). Hydrogen peroxide is scavenged by ascorbate-specific peroxidase in spinach chloroplasts. Plant Cell Physiol..

[49] Rao MV, Paliyath G, Ormrod DP (1996). Ultraviolet-B- and ozone-induced biochemical changes in antioxidant enzymes of Arabidopsis thaliana. Plant Physiol..

[50] Jackson ML (1973). Soil chemical analysis.

[51] Baruah TC (1997). A Textook of Soil Chemical Analysis.

[52] Livak KJ, Schmittgen TD (2001). Analysis of relative gene expression data using real-time quantitative PCR and the 2-ΔΔCT method. Methods.

