# Supplementary material for: Red-seaweed biostimulants differentially alleviate the impact of fungicidal stress in rice (Oryza sativa L.)
Source: Sci Rep. 2022 Apr 9;12:5993. doi: 10.1038/s41598-022-10010-8 (PMC8994781; doi:10.1038/s41598-022-10010-8)
Supplement: Supplementary file 1 — Supplementary Information 1. [file 41598_2022_10010_MOESM1_ESM.docx]

**Supplementary Figures**

**Supplementary Figure S1.** Quadratic regression analysis showing general statistical trends in (A) Potassium (B) Calcium (C) Temperature and (D) Stomatal closure.

**Supplementary Figure S2.** Quadratic regression analysis showing general statistical trends in (A) hydrogen peroxide (B) Super oxide radicals (C) Catalase activity (D) APX activity (E) SOD activity (F) POD activity and (G) GR activity.

**Supplementary Figure S3.** Quadratic regression analysis showing general statistical trends in (A) *APX,* (B) *E2F,* (C) *HSFA2A,* (D) *HSFB2B,* (E) *HSFB4C,* (F) *HSFC1A* and (G) *OsZIP-12*.
